# Transcatheter measurement of mitral valve coaptation pressure: A proof‐of‐concept study using animal models

**DOI:** 10.1002/btm2.70095

**Published:** 2025-11-25

**Authors:** Joseph Faudou, Anupriya Roul, Mohammed Benwadih, Minh‐Quyen Le, Anthony Medigo, Jean‐François Obadia, Pierre‐Jean Cottinet, Daniel Grinberg

**Affiliations:** ^1^ Université Grenoble Alpes, CEA, LITEN Grenoble France; ^2^ LGEF Laboratory, INSA Lyon, UR682 Villeurbanne France; ^3^ MitralPrint New York New York USA; ^4^ Department of Adult Cardiac Surgery “Louis Pradel” Hospital, Lyon Medical School Bron France

**Keywords:** animal study, force sensors, mitral valve coaptation, mitral valve repair

## Abstract

Mitral valve repair (MVr) is the preferred surgical treatment for primary mitral regurgitation; however, its success is limited by the lack of validated, accurate, and objective parameters for assessing the complete restoration of physiological mitral valve (MV) mechanics. Consequently, to address this challenge, intraoperative assessment of mitral valve coaptation pressure (MCP) has emerged as a promising approach. This study presents the first precise transcatheter measurement of MCP in animal hearts. Data were obtained using two custom‐made force sensors: a 3Fr piezoresistive pressure catheter and a 15Fr flexible piezoelectric sensor. Experiments were conducted in both ex vivo (excised pig hearts activated by a pump) and in vivo (transseptal approach in a living pig) models. In a living pig with a healthy MV under normal hemodynamic conditions (peak systolic left ventricular pressure of 100 mmHg), the MCP ranged from 200 to 300 mmHg (25–40 kPa). Ex vivo experiments demonstrated that MCP was affected by transmitral pressure, mitral function changes (i.e., regurgitation), and MV morphology. These findings provide valuable insights into MV biomechanics and establish a solid foundation for developing medical devices to guide MVr procedures.

## Graphical abstract

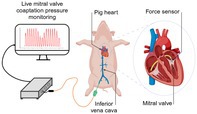

**Translational Impact Statement**

This study reports the first transcatheter measurement of mitral valve coaptation pressure in animal hearts, ranging from 200 to 300 mmHg, thereby laying the foundation for a novel medical device aimed at improving the success of mitral valve repair surgery.

## 1 INTRODUCTION

Mitral regurgitation (MR) is one of the most common valvular diseases, affecting approximately 2% of the general population, with its prevalence increasing significantly with age. It results from defective closure of the mitral valve (MV), causing the backflow of oxygenated blood (in the heart) from the left ventricle (LV) to the left atrium (LA). This condition can lead to life‐threatening complications, including heart failure and stroke.^1^, ^2^

Mitral valve repair (MVr) is the gold standard treatment for primary MR.^3^ In Western countries, long‐term outcomes are excellent and durable when performed by expert surgeons. However, repair rates and outcomes vary widely across health centers, depending on surgeon expertise, center volume, and experience.^4^ The average mortality rate is 3% during hospitalization, 6% after 1 year, and 10% after 5 years. Additionally, MR recurrence occurs in approximately 20% of patients within 5 years, following an initially successful repair.^5^, ^6^, ^7^

To improve the success rate and long‐term outcomes of MV surgery, several studies have focused on measuring the physical properties of MV to better understand its biomechanics. These experiments included the measurement of stress and strain in the annulus, chordae tendineae, and MV leaflets.^8^, ^9^, ^10^, ^11^, ^12^, ^13^, ^14^, ^15^ The devices and methods used in these studies provide a robust foundation for developing medical technologies that enable precise and objective intraoperative measurements, which help surgeons make informed decisions during surgery.^16^, ^17^

Recently, research on MV biomechanics has progressively shifted from physical measurements to finite element method (FEM) simulations, which use medical imaging (e.g., 3D echocardiography, magnetic resonance imaging or micro‐CT) to reconstruct patient‐specific geometries and model valve motion. In the long term, such simulations could non‐invasively recover mechanical parameters and ultimately predict surgical outcomes to optimize repair techniques.^18^, ^19^, ^20^, ^21^, ^22^, ^23^

Nevertheless, in vivo physical measurements remain essential to calibrate FEM simulations. While imaging‐based models can reproduce valve motion and deformations with high fidelity, the underlying stress distribution is more uncertain. The tissues of the MV exhibit complex, non‐elastic, and anisotropic mechanical behavior. Current simulations are based on ex vivo mechanical tests but generally do not account for variations in mechanical properties among individuals, particularly with age.^24^, ^25^ Moreover, some simulations use simplified conditions, either relying on echocardiography with limited geometric accuracy, or approximating blood flow as a uniform pressure field.^26^ For instance, under such conditions, Simonian et al. were compelled to introduce artificial force fields to match observed kinematics.^27^ Even studies using more complex models that account for fluid–structure interaction highlight the lack of experimental validation data and show discrepancies when compared to artificial MV models, although these are less complex than biological valves.^28^ This highlights the need for complementary experiments, like those provided in our study, to strengthen FEM‐based approaches.

Among all the components of the MV apparatus, leaflet coaptation has received considerable attention. This process, in which the anterior and posterior leaflets make contact during systole, is essential for the valve‐sealing mechanism. Numerous studies have shown that coaptation characterization, especially coaptation height (or length) measured postoperatively using echography, is a key factor for predicting short‐ and long‐term MVr success.^29^, ^30^, ^31^, ^32^, ^33^ However, few intraoperative assessments of coaptation have been developed, with most relying on visual observation.^34^, ^35^

Measuring mitral valve coaptation force—the contact force between leaflets during systole—is a promising approach for intraoperative assessment of the coaptation mechanism. Reportedly, coaptation force measurements can help differentiate regurgitation from a healthy valve, but these results have only been achieved using in vitro settings.^36^, ^37^ Recently, we demonstrated the feasibility of this measurement in vivo; however, we were unable to obtain clear and reliable force data.^17^

In this study, we present a new method to evaluate leaflet coaptation stress in animal hearts. Unlike previous literature that refers to coaptation force,^17^, ^36^ we define it as mitral coaptation pressure (MCP), that is, the contact force per unit area, normal to the leaflet surface. This terminology offers two advantages: it makes the value independent of the sensor's surface area and enables direct comparison with ventricular blood pressure.

MCP represents only the normal component of the leaflet contact stress, yet several arguments suggest it is the predominant one:

1. Coaptation is primarily driven by the transvalvular pressure difference, that is transmitted mainly perpendicularly to the leaflet surface.
2. Tangential interactions from friction are minimal between soft and wet tissues such as MV leaflets which have a friction coefficient of approximately 0.05. Therefore, these interactions are often neglected in mitral valve models.^38^, ^39^

Although MCP would remain an incomplete descriptor—fully characterizable only through computational analysis—it already provides clinically relevant information and a practical intraoperative parameter for surgeons.

**FIGURE 1:**
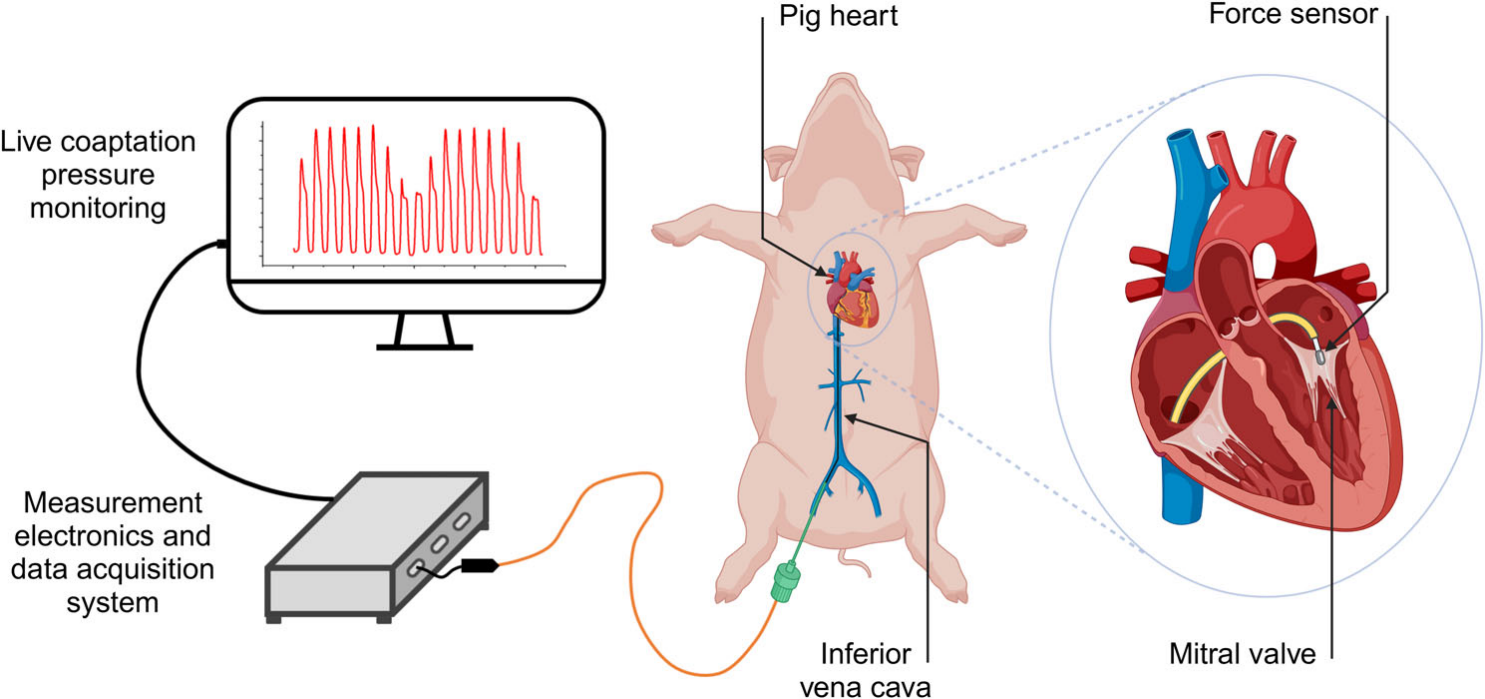
Illustration of in vivo MCP measurement in a pig using the transseptal approach.

Therefore, to achieve MCP analysis, we developed two custom‐made sensors to measure MCP during transcatheter mitral valve repair (TMVr) using a transseptal approach. For the first time, we measured MCP in a living animal under physiological conditions, as illustrated in Figure 1.

The test workflow consisted of two sets of experiments: (1) The sensors were first evaluated using an ex vivo model with direct access to the LA and controlled hemodynamic parameters. These controlled conditions enabled us to fully assess sensor performance and study key physical parameters, such as ventricular pressure, inter‐individual variation, and regurgitation. (2) The MCP was then measured in an in vivo pig model to obtain essential physiological data and assess the feasibility of measurement during a percutaneous transseptal MVr procedure.

## 2 RESULTS

### 2.1 Force sensors

**FIGURE 2:**
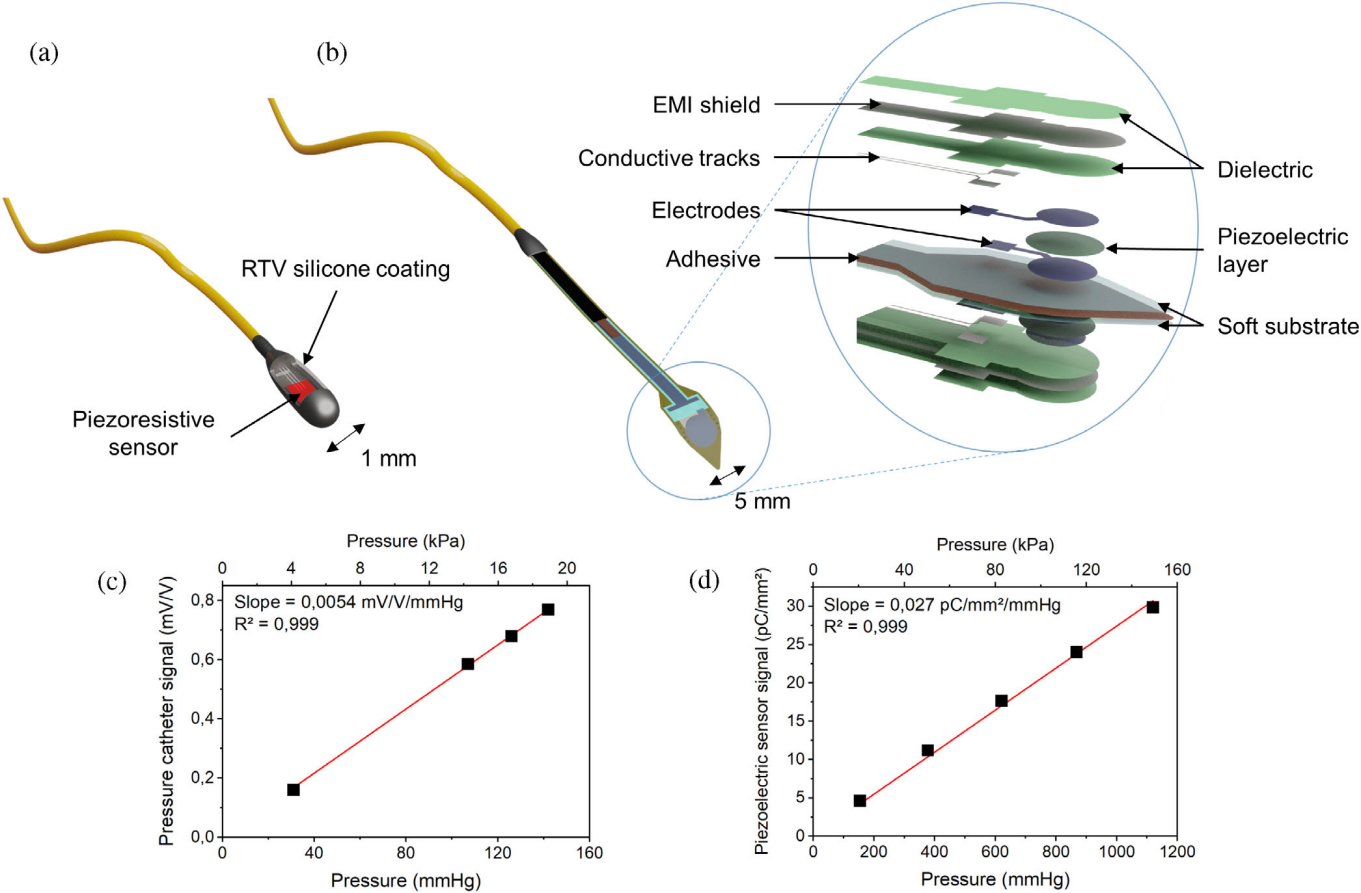
Force sensors used to measure MCP. Illustration of a (a) pressure catheter and a (b) flexible piezoelectric sensor. Calibration curves for the (c) pressure catheter and the (d) piezoelectric sensor.

Two types of force‐sensor technologies (Figure 2a,b) were used in this study. Using two different measurement methodologies helps address the limited points of comparison available in the literature.^36^, ^37^

The first sensor was a pressure catheter purchased from Millar, originally designed for blood pressure measurements and fractional flow reserve (FFR) procedures, as illustrated in Figure 2a. It is a 105‐cm long thin wire with a maximal diameter of 1 mm (3 Fr) and a piezoresistive sensing element at the bottom of the cavity as its tip. The device was slightly modified with a room‐temperature vulcanizing (RTV) silicone coating to fill the cavity, enabling the pressure catheter to measure force through direct contact with a solid object. Calibration was performed by measuring known pressures in a saline solution, yielding a clear linear signal (Figure 2c). This sensor offers a key advantage as a mature miniaturized technology suitable for in vivo transcatheter measurements. Its design enables the measurement of ventricular and atrial pressures in addition to MCP, and its linear shape allows MCP assessment along the coaptation height. However, two limitations may compromise the reliability of MCP measurements: (1) The coating must face a valve leaflet to obtain a signal, posing challenges in orientation; (2) the sensor was originally designed to measure pressure in fluids rather than direct contact force, making its mechanical interaction with the leaflets uncertain.

Thus, to overcome the limitations of the pressure catheter and improve measurement reliability, a second force sensor was investigated. As illustrated in Figure 2b, the proposed piezoelectric device consists of a conformable sheet with a double‐sided structure and electromagnetic interference (EMI) shielding. Designed and manufactured via screen printing, it is specifically tailored to measure the pressure applied on complex surfaces, such as soft biological tissues, making it a suitable reference for MCP measurement. The mechanical behavior of this sensor is well understood and was thoroughly investigated in a previous study.^40^ Its flexibility allows it to conform to the shape of the leaflets, ensuring that MV coaptation generates a compressive force on its surface. Its double‐sided structure eliminates the influence of bending stresses, due to conformability on the leaflets' surface, on the output signal. The sensor was calibrated by applying a known compressive force through direct contact with a solid object, yielding a clear linear signal (Figure 2d). Its flat structure could enable MCP mapping over the entire coaptation surface when several sensors are assembled in an array. However, this sensor remains a prototype, requiring further development to meet medical standards, particularly for minimally invasive cardiac surgery. With a maximum width of 5 mm, the piezoelectric sensor is larger than the pressure catheter, making it more suitable for traditional open‐heart surgery, such as sternotomy or minimally invasive mitral valve surgery (MIMVS). Nevertheless, its dimensions should still be compatible with certain surgical procedures that use a transseptal approach, such as the MitraClip procedure (Abbott).

The point‐by‐point comparison of the two sensors is summarized in Table 1.

**TABLE 1:** Comparison between pressure catheter and flexible piezoelectric sensor.

	Pressure catheter sensors	Flat flexible piezoelectric sensors
Technology	Piezoresistive Semi‐custom (coating on a commercial FFR guidewire)	Piezoelectric Fully custom‐made
Performance	Improve maneuverability	Good pressure measurement reliability
Use	Percutaneous transseptal mitral procedures	Reference sensor Conventional open heart MVr (sternotomy or MIMVS)
Measured parameters	Longitudinal coaptation force mapping (coaptation height) Simultaneous LA and LV pressure	2D coaptation force mapping (coaptation surface)

### 2.2 Ex vivo measurements in pig hearts

**FIGURE 3:**
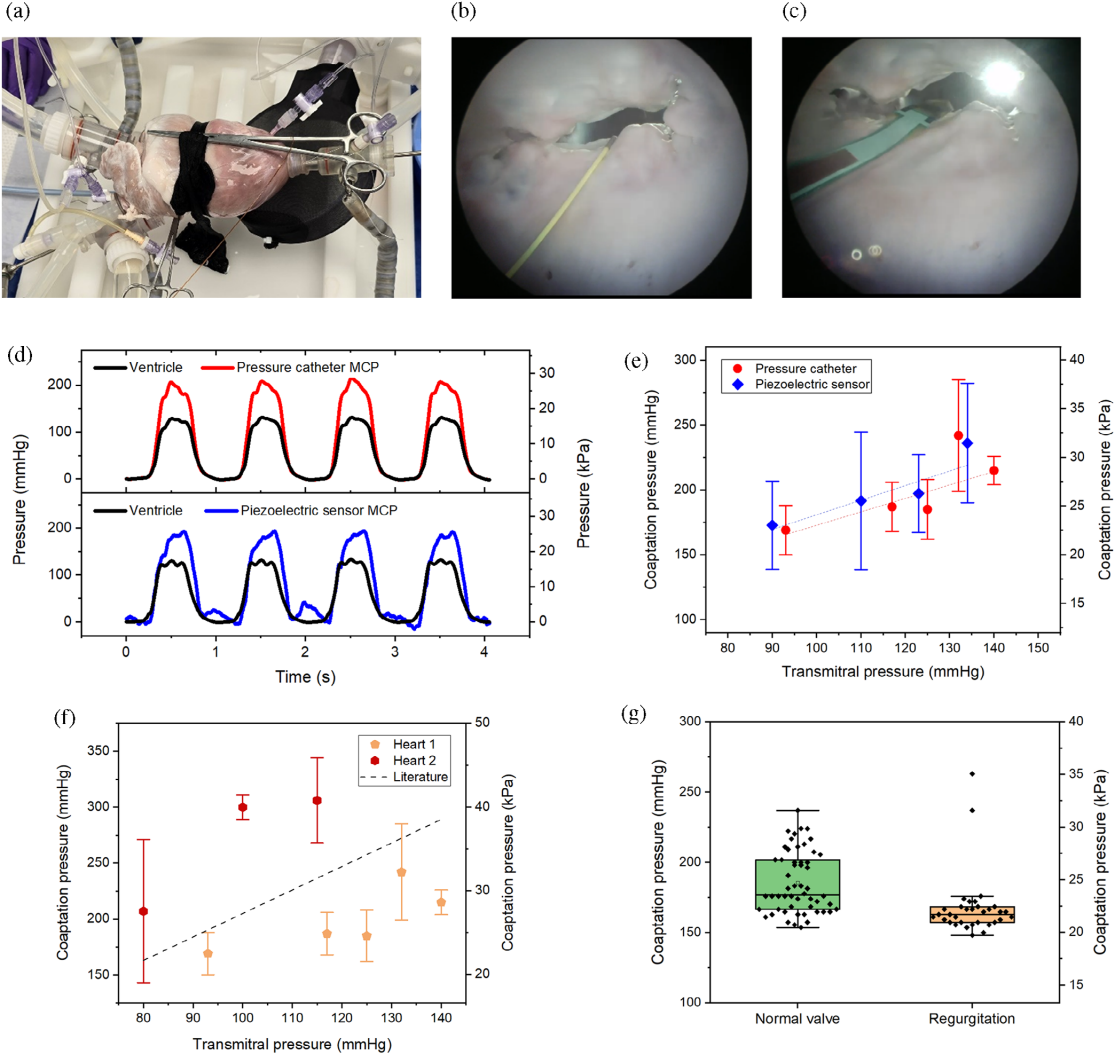
Results of ex vivo experiment. (a) Image of an excised pig heart in the biosimulation platform; (b) pressure catheter and (c) piezoelectric sensor in the MV; (d) temporal signals from both sensors synchronized with ventricular pressure; (e) comparison of the measured coaptation pressure by both sensors as a function of transmitral pressure; (f) comparison of coaptation pressure between two hearts; (g) comparison of coaptation pressure between a healthy valve and MV regurgitation.

An excised pig heart was mounted onto a cardiac biosimulation platform with an external pump to simulate realistic hemodynamic conditions and control pressure (Figure 3a). The peak systolic ventricular pressure was adjusted between 100 and 160 mmHg, with a diastolic pressure of 15–20 mmHg. Two endoscopes were positioned to provide top and bottom views of the MV. The left atrial and ventricular pressures were recorded, and both the pressure catheter and piezoelectric sensor were inserted transatrially (Figure 3b,c and Movies S1 and S2, Supporting Information).

With the aid of endoscopes and real‐time visualization of sensor signals, after a few minutes of practice, both sensors were successfully placed in a stable position with good contact with the valve leaflets in the A2‐P2 position. As shown in Figure 3d, the MCP signal was perfectly synchronized with the ventricular pressure signal. The MCP values were calculated using the calibration curves shown in Figure 2. Both sensors generated nearly identical results, with MCP exceeding the transmitral pressure. A clear correlation was observed between MCP and transmitral pressure (Figure 3e; *p* < 0.05)—higher transmitral pressure corresponded to higher coaptation pressure.

Two different hearts were studied, and the results are shown in Figure 3f. Both MVs exhibited similar MCP values, ranging from 150 to 300 mmHg (20–40 kPa). The pressure in the first heart was lower than that in the second heart (*p* < 0.001 for a transmitral pressure of approximately 115 mmHg). This behavior may be explained by anatomical observations (Figure S1). Overall, all data were comparable to in vitro measurements reported in the literature.^36^

The consistency between measurements from the two types of sensors and those from previous studies confirms the accuracy of our method. For subsequent experiments, the pressure catheter was preferred due to its miniaturized design, which facilitates insertion into the MV.

At the end of the experiment, MR was deliberately induced by cutting the chordae from the posteromedial papillary muscle, thus creating an A3‐P3 prolapse, as illustrated in Figure S2. Measurement of MCP during regurgitation was still performed at A2‐P2, but the reduced coaptation surface area made this considerably more challenging. Despite these difficulties, a reliable signal was eventually obtained and compared with that of a healthy valve, as shown in Figure 3g, at a similar transmitral pressure of 120 mmHg. MCP was significantly lower during regurgitation (186 ± 22 mmHg for a healthy MV versus 168 ± 23 mmHg for MR, *p* < 0.001).

### 2.3 In vivo measurements

**FIGURE 4:**
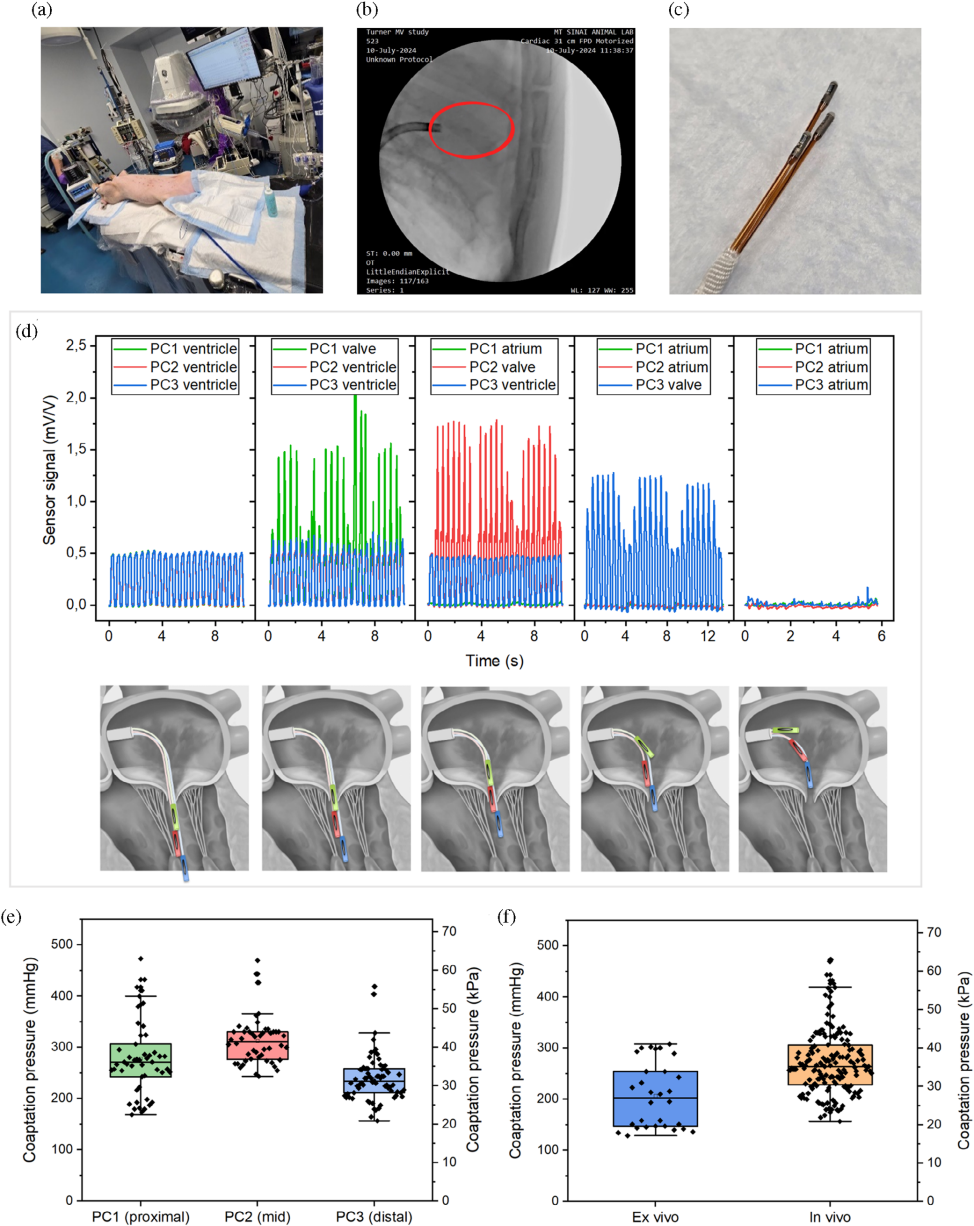
Results of in vivo experiment. (a) Image of the operating room; (b) fluoroscopy image of pressure catheters in the MV (sensors are barely visible in the red circle); (c) three pressure catheters assembled together; (d) signals from three pressure catheters (PCs) passing successively from the LV to the MV and then to the LA; (e) comparison of the coaptation pressure measured by the three pressure catheters; and (f) comparison of the coaptation pressure between in vivo and ex vivo experiments.

An in vivo experiment was conducted on a live pig model (Figure 4a), using a percutaneous transseptal approach to insert pressure catheters guided by fluoroscopy (Figure 4b). However, since positioning the sensor in the MV using X‐ray images is challenging, a complementary method was adopted to ensure accurate positioning. Three pressure sensors were assembled with slight millimeter‐scale spacing between them (Figure 4c). They were pushed into the LV, then slowly retracted through the MV and placed in the LA. Because ventricular and atrial pressure signals were easily distinguishable, the successive passage of these sensors through the MV was identified and validated for correct positioning, as illustrated in Figure 4d.

An interesting observation was the presence of two distinct frequencies in the signal from the sensor in the MV, as shown in Figure 4d, and further demonstrated by the fast Fourier transform of the signal presented in Figure S3. The primary frequency at approximately 115 bpm corresponded to the heart rate, while the secondary signal at a lower frequency was associated with breathing (13 breaths/min). This finding indicates that respiratory movement affects MCP measurements. The variations in signal shape suggest that, at certain points in the respiratory cycle, the sensor temporarily shifts from the MV to the LV before returning.

The pressure catheter signal recorded during the in vivo experiment was significantly higher than that in the ex vivo experiment. The amplitudes of some signals exceeded the measurable pressure range recommended by the manufacturer, leading to inconsistent signals. As a result, approximately 25% of the data was excluded using the statistical approach described in Figure S4. Therefore, the MCP values estimated from the remaining data were slightly underestimated.

Figure 4e shows that all three sensors measured MCP in the same range of 200–350 mmHg (25–45 kPa). However, notable differences were observed, particularly for the sensor in the distal position, which recorded lower pressures. These variations likely resulted from differences in measurement locations on the MV, as precise positioning could not be fully controlled. Additionally, some sensors may not have been perfectly aligned with the leaflets, leading to an underestimation of the measured pressure.

The coaptation pressures measured ex vivo and in vivo at a similar transmitral pressure of approximately 80 mmHg are compared in Figure 4f. While both were measured within the same range, the MCP was significantly higher in the in vivo test (207 ± 64 mmHg ex vivo vs. 266 ± 70 mmHg in vivo, *p* < 0.001).

## 3 DISCUSSION

Overall, the MCP was measured in the range of 200–300 mmHg (25–40 kPa) and was higher in the in vivo heart than in the ex vivo model. This difference was expected, given the “reversed” physiologic concept of the two models: under in vivo conditions, the heart contracts during systole, while during ex vivo tests, as the beat is induced by an external pump, the heart expands during systole. This results in differences in annulus contraction between the two models, which may have contributed to the discrepancy.

Notably, MCP was consistently measured as higher than the transmitral pressure, raising an interesting question: How can a heart generating a ventricular pressure of 120 mmHg produce a MCP of 300 mmHg? Our main hypothesis is that the complex MV apparatus concentrates mechanical stress at the coaptation level. In the coaptation plane, both leaflets press against each other, dissipating energy from the entire mitral apparatus. These high coaptation forces during systole ensure strong closure of the mitral orifice. Under pathological conditions, even a slight mechanical imbalance between the annulus, leaflets, and chordae in the MV can disrupt this stress absorption mechanism, increasing the risk of MV damage, such as chordal elongation or leaflet tearing, eventually leading to regurgitation. This is evident in our findings: despite hemodynamic parameters being similar to those of a healthy valve, MCP was lower during regurgitation than in a healthy valve, suggesting that mechanical stresses were redistributed elsewhere in the mitral apparatus. These insights offer new perspectives for understanding MV biomechanics.

These hypotheses still need to be validated by combining MCP measurements with other physical parameters in the mitral valve, such as chordae tension or leaflet strain, to gain a clearer picture of stress redistribution during regurgitation. Ultimately, FEM simulations could be informed by experimental data to infer the full biomechanical behavior of the MV. Interestingly, a convergence between measured and simulated data is already emerging. For example, Rim et al. reported simulated coaptation pressures in the range of 50–60 kPa (375–400 mmHg, slightly above our measurements), with a maximum that decreases when regurgitation occurs.^41^ These observations are consistent with our results.

Furthermore, our study has identified several factors influencing coaptation pressure. First, the MCP increases with the transmitral pressure, a phenomenon that is intuitive, expected, and consistent with in vitro tests in the literature.^36^ Moreover, MCP may vary slightly between hearts, due to natural differences in MV anatomy among individuals. This raises uncertainty as to whether it is possible to precisely identify a “nominal” MCP value which is representative of proper MV closure. Instead, we demonstrated that MCP decreases during MR, suggesting that variations in MCP could serve as a reliable indicator of MV health during surgery.

However, despite these promising results, several challenges remain before the application of this method to human health. Proper sensor positioning in the valve to obtain a reliable signal is difficult, even with the guidance of endoscopes. This is even more complicated when using fluoroscopy or in cases of regurgitation. Moreover, external factors, such as breathing movements, can interfere with measurements. These findings open new perspectives for developing a force sensor that is fully adapted for MV applications. They provide a first estimate of in vivo MCP, providing a basis for adapting the measurement range of the sensor. This would avoid bias due to the exclusion of certain outliers, as we obtained during the in vivo experiment. The optimal sensor design should combine the miniaturization of the pressure catheter with the measurement reliability and absence of orientation issues of the piezoelectric sensor. Additionally, improved navigation methods should be developed to ensure precise sensor positioning in the MV, such as the approach using three pressure catheters in the in vivo experiment. Obviously, the developed sensor and methods will then have to comply with all applicable regulations.

Furthermore, additional in vivo measurements should be carried out on several animals. This would validate the results of this study by improving statistical robustness.

## 4 MATERIALS AND METHODS

Animal experiments were approved by the Institutional Animal Care and Use Committee (IACUC, ID: IPROTO202200000119).

### 4.1 Pressure catheter

Pressure catheters were purchased from Millar (Pearland, TX). The manufacturer modified the commercial product by adding a thick RTV silicone coating above the sensor to facilitate contact force transmission. Pressure catheter signals were measured using a Wheatstone bridge circuit (with 4.7 kΩ resistors and a 2 V power supply), connected to a data acquisition (DAQ) system (Sirius from Dewesoft) with a 10 Hz low‐pass filter to reduce measurement noise. They were calibrated on the day of the ex vivo experiment by measuring ventricular pressure and using the values provided by the pressure transducers (included in the biosimulation platform) as references.

### 4.2 Piezoelectric sensors

Piezoelectric sensors were screen‐printed in a clean room on a soft 100‐μm‐thick thermoplastic polyurethane (TPU) substrate from Dupont. The active piezoelectric material was a 3‐μm layer of polyvinylidene fluoride trifluoroethylene (P(VDF‐TrFE)) with 80/20 proportion, purchased from Arkema Piezotech and poled with a 100 V/μm electrical field to activate its piezoelectric properties. Electrodes and EMI shielding are made of a 1‐μm‐thick poly(3,4‐ethylenedioxythiophene)‐poly(styrenesulfonate) (PEDOT:PSS) conductive polymer sourced from Heraeus. The non‐active dielectric material, used for a 6‐μm‐thick isolation layer and a 3‐μm‐thick encapsulation layer, consisted of unpoled P(VDF‐TrFE). Two sensors were assembled to form a piezoelectric bimorph by bonding their substrates with a 25‐μm polyimide substrate using VHB adhesive from 3M on each side. This double‐sided structure allowed measurement of the compressive force while eliminating contributions from bending stresses. The active‐sensor surface measured 8 mm^2^.

The piezoelectric response was measured using a DSI charge amplifier (Dewesoft) connected to the DAQ system, with a 10 Hz low‐pass filter to reduce noise and a 0.06 Hz high‐pass filter to prevent charge derivation. The sensors were calibrated by applying a uniform compressive load using a flat indenter attached to a micrometric motor.

Further details on the manufacturing, calibration, performance, and operating principles of the piezoelectric sensor are available in our previous study.^40^

### 4.3 Ex vivo experiment

An excised pig heart was mounted onto Resolution Medical's cardiac biosimulation platform, with a 1 Hz external pump simulating realistic hemodynamic conditions with a standard heart rate. Two pressure transducers monitored pressure in the LV and LA. A constraining fabric support layer was placed around the heart to prevent excessive systolic inflation, mimicking the supportive function of the pericardium. Two endoscopes were positioned to visualize the MV, one in the LV and the other in the LA. Sensors were inserted transatrially, with pressure catheters placed using an 8 Fr introducer sheath and piezoelectric sensors inserted through an 18 Fr sheath. A pressure catheter inserted through the apex remained in the LV as a reference sensor, and was synchronized with the sensor in the MV throughout the experiment.

Two of each sensor type were tested on two normal excised hearts. Guided by endoscopes, the sensors were precisely positioned between the MV leaflets, with the pressure catheter oriented to face the leaflets. The flat design of the piezoelectric sensor indicated that its orientation was not a concern. Each sensor was advanced through the valve into the ventricle and slowly retracted into the MV. Synchronization of the signal with ventricular pressure allowed detection of the sensor position and signal changes within the MV. Measurements were taken at the A2‐P2 location of the MV while the pump was controlled to adjust hemodynamic conditions. The sensor signal was recorded across a range of left ventricular pressure from 100 to 160 mmHg. The transmitral pressure, estimated as the pressure difference between the LA and LV across the MV, varied between 80 and 140 mmHg. For one heart, to induce MR, chordae at the posteromedial papillary muscle were cut using endoscopic scissors, and the experiment was repeated.

For practical reasons (time restrictions for the experiment due to heart inflation over time, and the ability to keep the sensor in a stable position in the MV), each measurement lasted approximately 30 s before changing conditions.

### 4.4 In vivo experiment

The in vivo experiment was conducted in an animal laboratory at Mount Sinai Hospital. The tests were carried out on a single animal, as the objective was to demonstrate proof of concept of the method rather than to conduct a full preclinical study on a cohort. The animal used was a 3‐month‐old male pig weighing 39 kg. At this stage of our research, the precise characteristics of the animal are not determining factors.

A femoral vein puncture was performed, and a needle was inserted through the inferior vena cava into the right atrium to achieve percutaneous transseptal puncture and access the LA under fluoroscopic guidance. A long curved sheath was positioned above and centered over the MV in the LA to hold the device (LA sheath). This route was chosen because it is the least traumatic for accessing the MV and is commonly used during MVr procedures. The pressure catheters were pushed through the LA sheath into the MV opening and into the LV. Larger piezoelectric sensors were not used because the vascular anatomy of pigs is not suitable for catheters of this size. Guided by fluoroscopy and continuous monitoring of vital signs, the ventricular pressure was recorded to estimate transmitral pressure, which ranged from 80 to 90 mmHg.

Owing to the limited visibility of the sensor in the MV (Figure 4b) and the difficulty in controlling its orientation, three pressure sensors were assembled with slight millimeter‐scale spacing (see Figure 4c). All the sensors were inserted through the sheath, advanced into the LV, and gradually retracted across the MV into the LA. In this setup, the distal sensor detected the peaks first, followed by the middle and proximal sensors. While sensor orientation remained random, one of the four experiments (on a single pig) achieved successful alignment, with all three sensors facing the MV leaflet.

As breathing affected the sensor signal, only the peaks with the highest amplitude during the breathing cycle were used for analysis.

Each measurement (i.e., the measurement of one MCP with one sensor) was repeated two times and lasted approximately 30 s due to time restrictions and the ability to keep the sensors in a stable position.

Data analysis, including exclusion of measurements outside the sensor measurement range, was performed after the experiment. Outliers were clearly identified as their amplitude formed a Gaussian distribution outside the recommended measurement range, distinct from the distribution of correct measurements (Figure S4).

### 4.5 Statistical analysis

Each MCP measurement is a mean value over the sample and error bars represent standard deviations. The average sample size was 30 values, with each sample representing a single coaptation pressure using one sensor at a given transmitral pressure. Each value corresponded to the pressure amplitude of one cardiac cycle. Sample size therefore corresponds approximately to the number of successive cardiac cycles in which the sensor was in the MV. Sample size was constrained by practical considerations, notably the limited duration of the experiments and the ability to keep a sensor in a stable position in the MV. No power analysis could be performed to estimate the appropriate sample size prior to the experiments as no MCP measurements had previously been carried out on animals.

*P*‐values for pressure comparisons were calculated using a two‐sample *t* test, considering normal distributions. The *p*‐value used to evaluate the correlation between coaptation pressure and transmitral pressure was calculated using Pearson's correlation coefficient.

## 5 CONCLUSION

To our knowledge, this is the first transcatheter measurement of MCP in a living animal under physiological conditions. Ex vivo measurements in an excised heart validated the reliability of the technique by comparing two different sensors, and enabled the identification of various factors that can influence coaptation. The feasibility of this measurement was further demonstrated in vivo during a percutaneous transseptal procedure in a pig.

Overall, the MCP was measured in the range of 200–300 mmHg (25–40 kPa) in a healthy valve, and was lower during regurgitation (150–200 mmHg). Moreover, it was consistently higher than the transmitral pressure (80–120 mmHg), while remaining correlated with it.

These findings provide deeper insight into the biomechanical balance within the mitral apparatus and suggest that intraoperative MCP measurement could be a valuable tool during MVr. It may serve as a good indicator of repair effectiveness and help predict the likelihood of recurrent regurgitation.

This study lays the foundation for the development of a medical device to measure MCP during MVr. Ongoing developments in force sensor technology aim to improve the reliability and ease of measurement in future in vivo animal trials.

## AUTHOR CONTRIBUTIONS

*Conceptualization*: Daniel Grinberg, Pierre‐Jean Cottinet, Anthony Medigo. *Methodology*: Daniel Grinberg, Joseph Faudou, Anupriya Roul, Anthony Medigo. *Investigation*: Joseph Faudou, Anupriya Roul, Daniel Grinberg, Mohammed Benwadih. *Visualization*: Joseph Faudou, Anupriya Roul, Daniel Grinberg. *Funding acquisition*: Anthony Medigo, Pierre‐Jean Cottinet, Daniel Grinberg. *Project administration*: Anthony Medigo, Pierre‐Jean Cottinet, Daniel Grinberg. *Supervision*: Daniel Grinberg, Pierre‐Jean Cottinet, Anthony Medigo, Jean‐François Obadia. *Writing – original draft*: Joseph Faudou, Anupriya Roul. *Writing – review and editing*: Minh‐Quyen Le, Daniel Grinberg.

## FUNDING INFORMATION

National Institutes of Health (NIH) Phase I Small Business Technology Transfer (STTR), Grant Number: 1R41HL167401‐01. French Alternative Energies and Atomic Energy Commission (CEA).

## CONFLICT OF INTEREST STATEMENT

The authors declare no conflicts of interest.

## Supporting information


**Data S1.** Supporting Information.

**Movie S1.** Pressure catheter in the mitral valve during ex vivo testing.

**Movie S2.** Piezoelectric sensor in the mitral valve during ex vivo testing.

## Data Availability

The data that support the findings of this study are available from the corresponding author upon reasonable request.
