# Supplementary material for: Transcatheter measurement of mitral valve coaptation pressure: A proof‐of‐concept study using animal models
Source: Bioeng Transl Med. 2025 Nov 25;11(1):e70095. doi: 10.1002/btm2.70095 (PMC12821221; doi:10.1002/btm2.70095)
Supplement: Supplementary file 1 — Data S1. Supporting Information. [file BTM2-11-e70095-s003.docx]

**SUPPLEMENTARY MATERIALS**

**
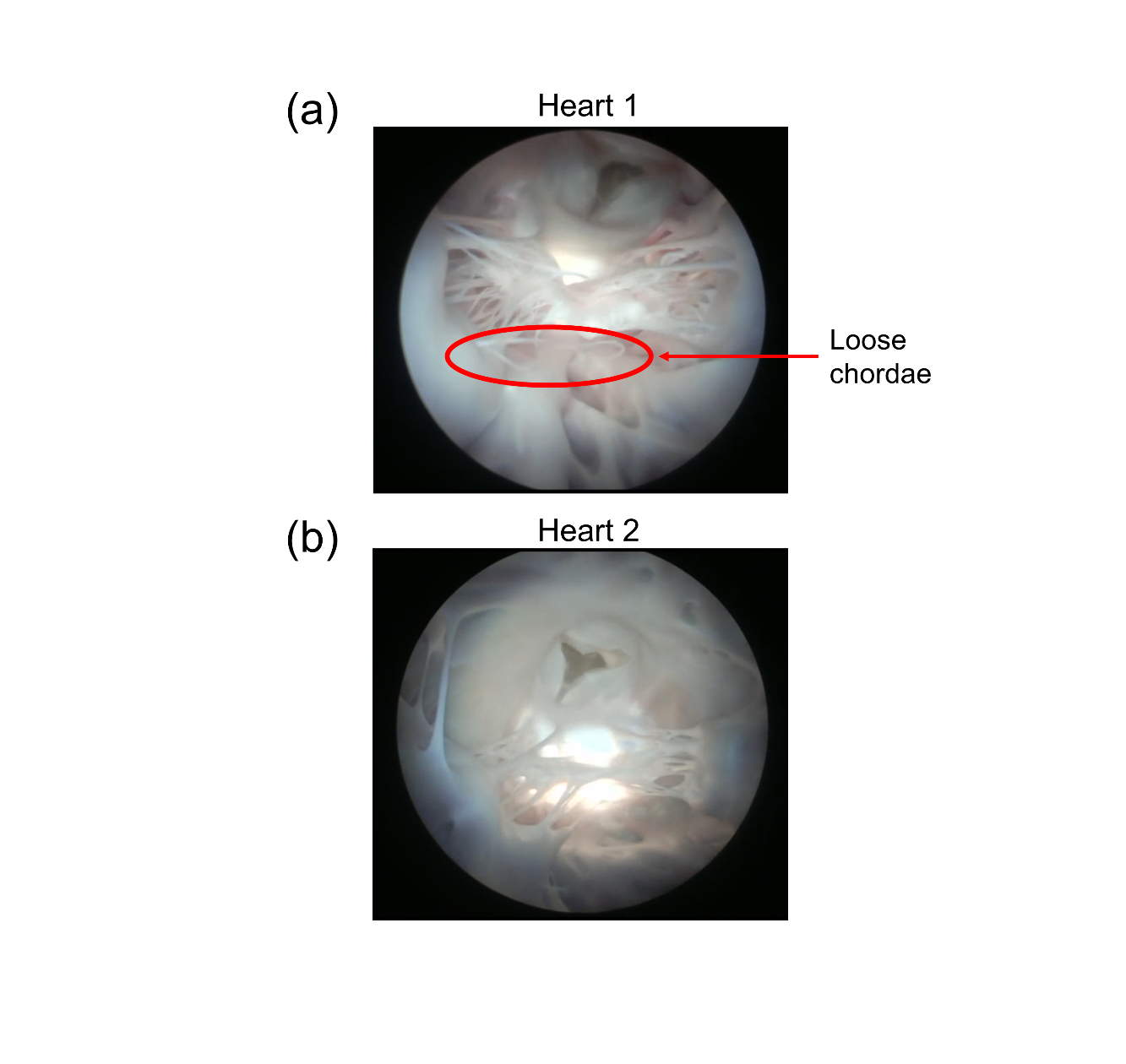
**

Figure S1. Morphological observation of the mitral valve (MV) from the left ventricle during systole. (a) First heart. (b) Second heart. Chordae are expected to be under tension during systole to ensure proper MV closure. The observation of loose chordae in the first heart can explain the slightly weaker closure, and consequently, lower coaptation pressure.

**
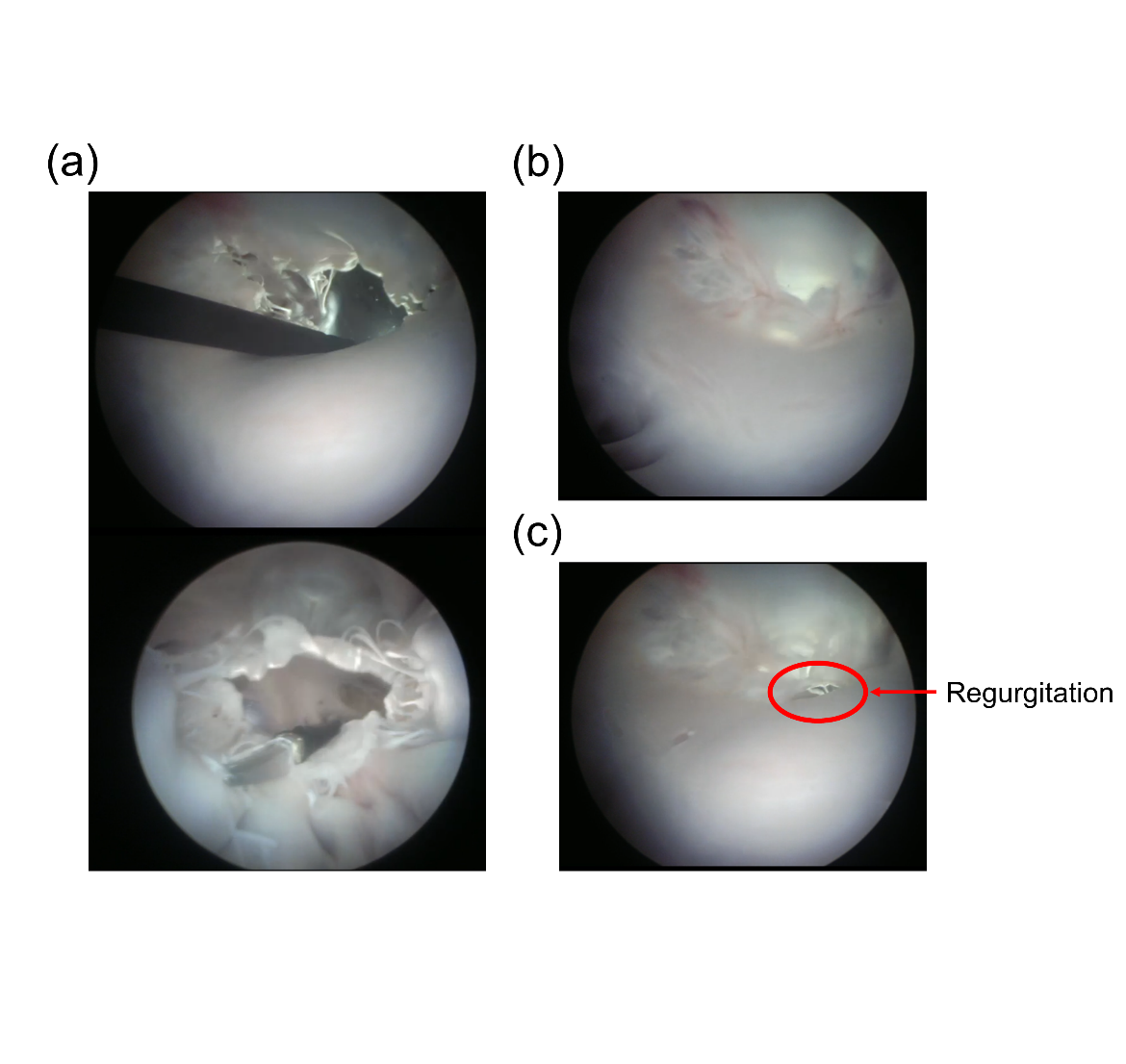
**

Figure S2. Induction of mitral regurgitation. (a) Endoscopic scissors are used to cut the chordae at the posteromedial papillary muscle to induce mitral regurgitation. Observation of the MV from the left atrium during systole in (b) a healthy valve and (c) valve with mitral regurgitation.


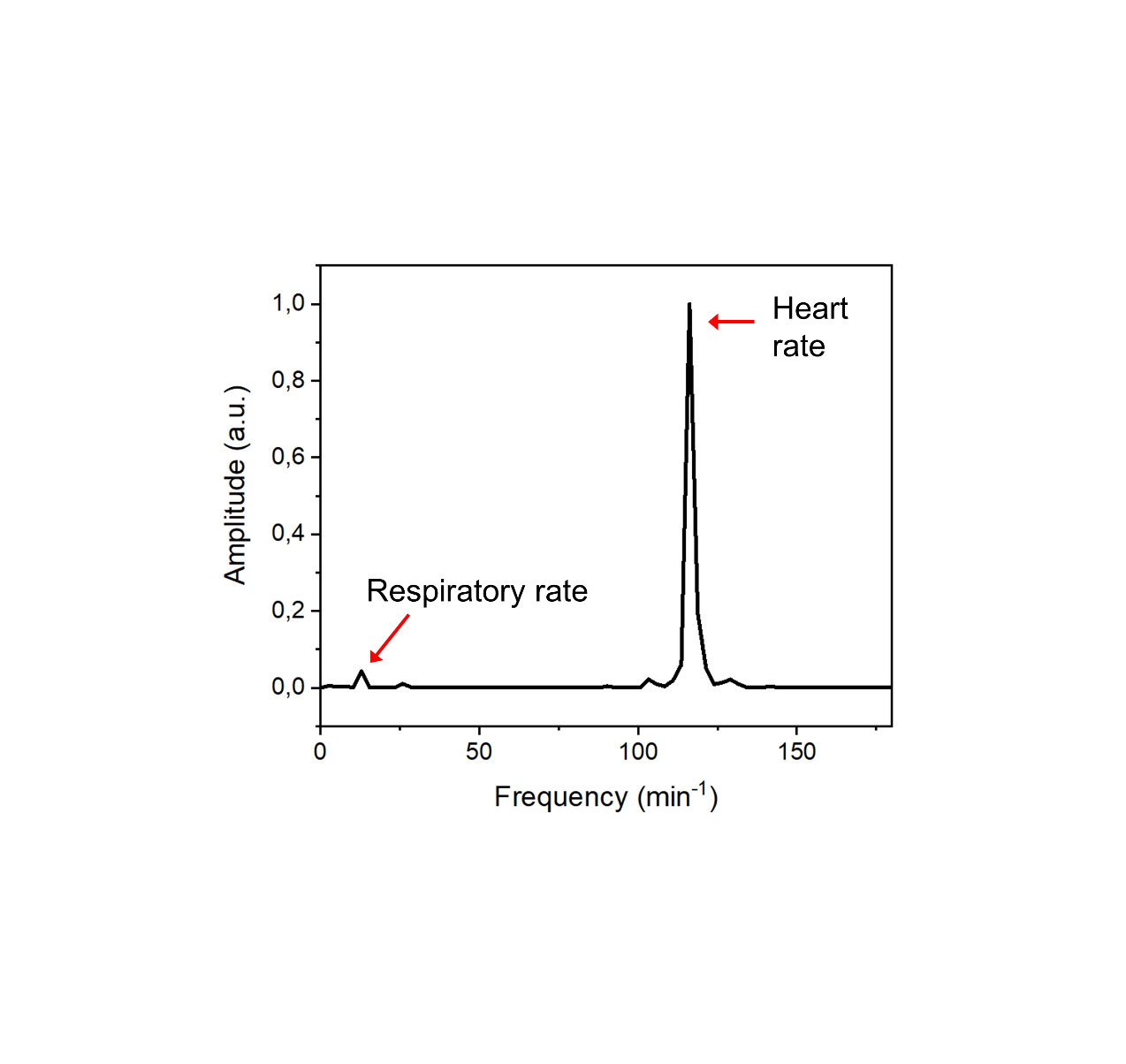


Figure S3. Fast Fourier transform of in vivo coaptation pressure signal. The analysis reveals that the respiratory rate introduces disturbances in the signal.


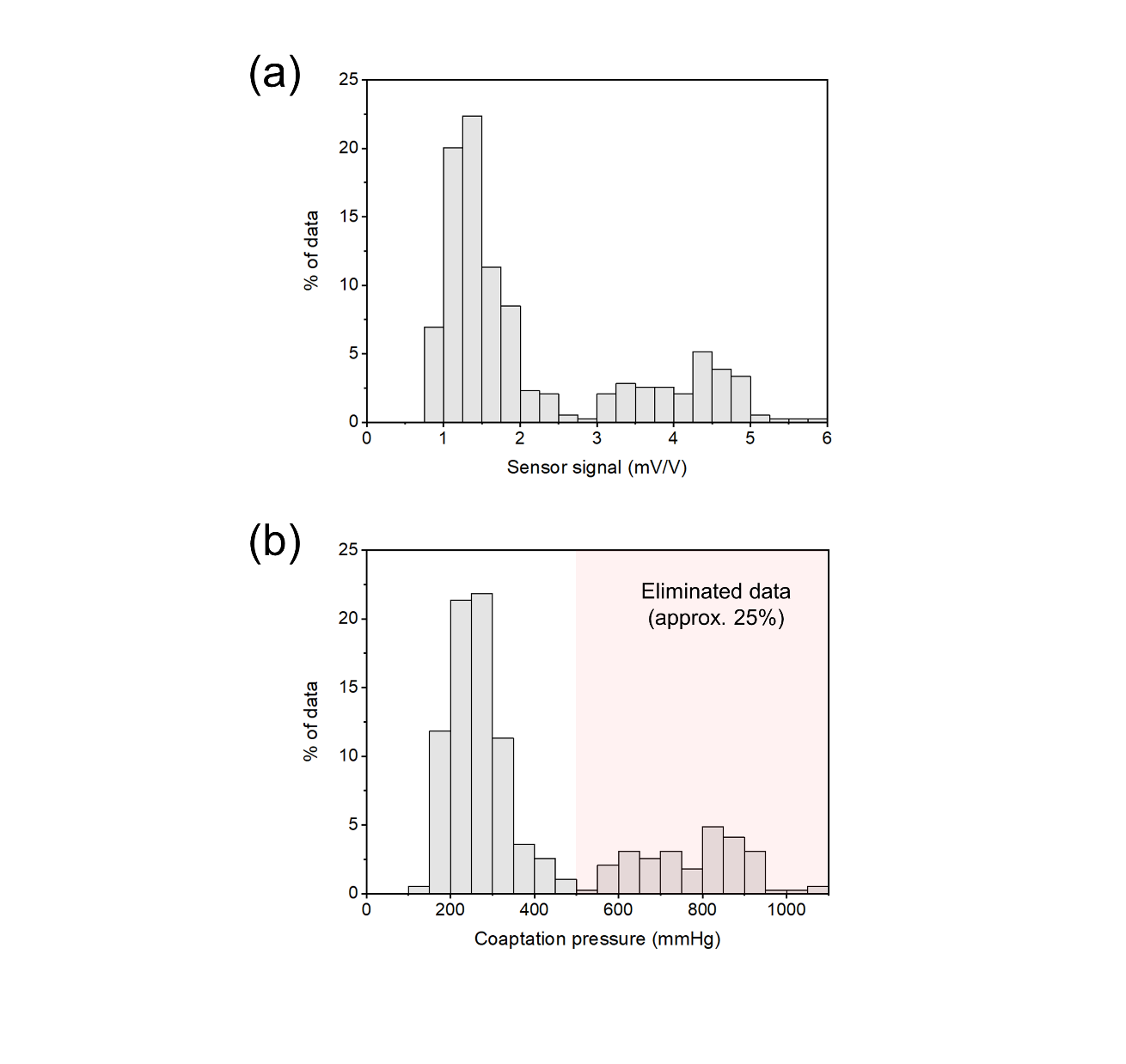


Figure S4. Amplitude distribution of pressure catheter signal during the in vivo experiment. (a) Distribution of maximum peak amplitude. (b) Distribution of calculated coaptation pressure. Despite measuring the same phenomenon, two distinct sensor behaviors are identified, resulting in two separate amplitude distributions: a low-amplitude distribution (between 1 and 2 mV/V), similar to values obtained in the ex vivo experiments, and a high-amplitude distribution (between 3 and 5 mV/V). The measurement range recommended by the manufacturer is –250 to +250 mmHg (relative to the atmospheric pressure). However, the high-amplitude distribution corresponds to pressures between 500 and 1000 mmHg—far surpassing the sensor’s capacity and inconsistent with expected pressure values in the heart. As the coaptation pressure is already near the sensor’s measurement limit, excessively high pressure peaks likely cause sensor saturation, leading to inconsistent behavior and deviations from calibration. Consequently, these high-pressure values are deemed false. Therefore, data from high-amplitude distribution are excluded in the coaptation pressure analysis.
